# Machine learning reveals neutrophil-to-lymphocyte ratio as a crucial prognostic indicator in severe Japanese encephalitis patients

**DOI:** 10.3389/fneur.2023.1242317

**Published:** 2023-12-20

**Authors:** Yaxuan Wei, Ying Hao, Yuanming Li, Meiling Dan, Zhiqi Yang, Huihui Qiu, Rong Li, Rong Yin, Pengcheng Fan

**Affiliations:** ^1^Department of Neurology, The Second Hospital of Lanzhou University, Lanzhou, China; ^2^Department of Neurology, Gansu Province Central Hospital, Lanzhou, China; ^3^Department of Neurology, Lanzhou General Hospital, Lanzhou, China; ^4^School of Life Sciences, Beijing University of Chinese Medicine, Beijing, China; ^5^Department of Neurological Rehabilitation, Sichuan China 81 Rehabilitation Center, Chongqing, China; ^6^The First Clinical Medical School, Gansu University of Chinese Medicine, Lanzhou, China; ^7^Department of Pharmacy, Lanzhou General Hospital, Lanzhou, China; ^8^State Key Laboratory of Proteomics, National Center for Protein Sciences, (Beijing), Institute of Lifeomics, Beijing, China

**Keywords:** Japanese encephalitis, neutrophil-to-lymphocyte ratio, percentage of neutrophilic, machine learning, cognitive impairment

## Abstract

Japanese encephalitis (JE) is a severe infectious disease affecting the central nervous system (CNS). However, limited risk factors have been identified for predicting poor prognosis (PP) in adults with severe JE. In this study, we analyzed clinical data from thirty-eight severe adult JE patients and compared them to thirty-three patients without organic CNS disease. Machine learning techniques employing branch-and-bound algorithms were used to identify clinical risk factors. Based on clinical outcomes, patients were categorized into two groups: the PP group (mRs ≥ 3) and the good prognosis (GP) group (mRs ≤ 2) at three months post-discharge. We found that the neutrophil-to-lymphocyte ratio (NLR) and the percentage of neutrophilic count (N%) were significantly higher in the PP group compared to the GP group. Conversely, the percentage of lymphocyte count (L%) was significantly lower in the PP group. Additionally, elevated levels of aspartate aminotransferase (AST) and blood glucose were observed in the PP group compared to the GP group. The clinical parameters most strongly correlated with prognosis, as indicated by Pearson correlation coefficient (PCC), were NLR (PCC 0.45) and blood glucose (PCC 0.45). In summary, our findings indicate that increased serum NLR, N%, decreased L%, abnormal glucose metabolism, and liver function impairment are risk factors associated with poor prognosis in severe adult JE patients.

## 1 Introduction

Japanese encephalitis (JE) is a severe form of viral encephalitis characterized by acute inflammation of the central nervous system, primarily caused by the Japanese Encephalitis Virus (JEV) (1). Although the development of JE occurs in only a small percentage of JEV-infected individuals (0.1–1%), it still poses a significant public health burden, with approximately 68,000 to 96,000 reported cases and 15,000 deaths worldwide each year (2, 3). Among severe JE cases with neurological dysfunction, the fatality rate can reach 20–52%, and neurological sequelae have been reported in 44–60% of patients (2). Furthermore, nearly 50% of JE patients continue to experience neurological sequelae even 1 year after hospital discharge (4). Conducting clinical studies with large sample sizes is challenging due to the regional and seasonal incidence patterns of JE.

To date, limited information is available regarding the risk factors associated with poor prognosis in severe adult JE cases. The Japanese Encephalitis Virus can directly induce neuronal damage, and the resulting inflammation can exacerbate this effect (5). Previous studies have indicated a correlation between the innate immune response and fatal outcomes in flavivirus infections (6). However, the major risk factors contributing to poor prognosis in JE infections remain unclear. Our previous research focused on analyzing proteome profiling changes in the cerebrospinal fluid (CSF) of severe adult JE patients, identifying a subgroup with lower survival rates and a higher risk of cognitive impairment (7). Identifying key factors associated with poor prognosis through clinical indicators is an ongoing research topic in JE.

In the past, the lack of extensive samples and high-quality clinical studies has hindered a clear understanding of the specific clinical indicators associated with adult JE. The onset and progression of adult JE are characterized by rapidity, while the prognosis remains unfavorable. How to utilize commonly employed clinical indicators for early prediction of unfavorable prognosis, thereby enabling prompt implementation of targeted therapeutic interventions such as early tracheotomy and mechanical ventilation, holds the potential to significantly mitigate both mortality and disability rates.

Machine learning has emerged as a valuable tool in investigating clinical risk factors. Branch and bound algorithms, specifically designed for logistic regression analysis using R script packages, are commonly employed for analyzing large-scale biomedical data (8). The present study aims to employ machine learning techniques and statistical analysis tools to identify risk factors for poor prognosis in severe adult JE patients using extensive clinical data. By leveraging these advanced analytical approaches, we seek to gain valuable insights into the prognosis of severe JE and contribute to the understanding and management of this debilitating disease. To the best of our knowledge, this study represents the pioneering application of machine learning in analyzing risk factors associated with unfavorable outcomes in adults affected by JE.

## 2 Materials and methods

### 2.1 Study design and setting

The study received approval from the Ethics Committee of Lanzhou General Hospital prior to initiation (2017XYLL050) and utilized data from the Chinese Clinical Trial Registration Study (ChiCTR2000030499). The participants were admitted to the hospital between July 2017 and December 2019. A total of thirty-eight patients diagnosed with JE during the acute stage of encephalitis were included in the study. These JE cases were reported to the Gansu Sub-center of the Center for Disease Control in China. As a control group, thirty-three patients who were hospitalized in the neurology department during the same period but excluded from having organic disease of the central nervous system (CNS) were enrolled. Machine learning analysis utilizing branch-and-bound algorithms was employed to select clinical risk factors from the data of JE patients and the control group. The criteria for defining JE were based on the World Health Organization recommendations (9), requiring the presence of clinical criteria for acute encephalitis syndrome and satisfying at least one of the following: detectable JE-specific IgM in the cerebrospinal fluid (CSF) or serum, evidence of seroconversion or a fourfold increase in IgM or IgG during the convalescence phase as detected by ELISA, isolation of the virus from blood, CSF fluid, or tissue, or detection of the JE virus genome in the serum, plasma, blood, CSF, or tissue. Information extracted from the database included demographic baseline data, vital signs, clinical symptoms, positive signs, endotracheal intubation or tracheotomy, complications and comorbidities, blood and CSF laboratory indicators, Glasgow Coma Score (GCS), Mini-Mental State Examination (MMSE) at discharge, and modified Rankin Scale (mRS) scores collected 3 months after discharge.

### 2.2 Machine learning analysis for JE infection risk factors

The selection of risk factors was conducted through logistic regression, combining best subset selection and cross-validation approaches for model selection (Supplementary Figure S1). A total of 45 clinical test results were collected to perform statistic analysis. Among them, 26 major clinical test results with statistic significant difference between 38 good and poor prognosis patients of JE were chosen as input factors. These included 4 CSF test results (RBC count, WBC count in CSF, Platelet Count, and pressure) and 22 blood test results (WBC, total protein, RBC, blood glucose, Cl, WBC in blood, percentage of neutrophilic count (N%), percentage of lymphocyte count (L%), percentage of monocyte (M%), hemoglobin, hematocrit, Platelet Count, lactate dehydrogenase, aspartate transaminase, alanine transaminase, gamma-glutamyl transferase, K^+^, Na^+^, Cl^−^, blood urea nitrogen, and creatinine). Branch-and-bound algorithms were implemented using the R script package “bestglm” which represents Best Subset GLM. The analysis utilized Bayesian Information Criterion with Bernoulli prior (“BICq”) as the information criteria.

### 2.3 Subgroups of patients with JE according to prognosis

Patients with JE were grouped according to the WS214-2008 Diagnostic Criteria for JE (10). All patients included in the study were classified as severe or critically ill. Prognosis-based categorization was performed 3 months after discharge, considering mRS ≤ 2 as the good prognosis group (GP) (*n* = 29) and mRS ≥ 3 as the poor prognosis group (PP) (*n* = 9).

### 2.4 Statistical analysis and data visualization

Continuous variables were expressed as mean (standard deviation) for normally distributed data and median (interquartile range [IQR]) for non-normally distributed data. One-way ANOVA test was conducted for data that adheres to a normal distribution. Non-parametric test (Wilcoxon rank-sum test) was performed for data that did not conform to a normal distribution. Enumeration data was presented as percentages and analyzed by the Chi-square test. A two-sided *p*-value of less than 0.05 was considered statistically significant. Data analysis and visualization were performed using R version 4.1.3. Principal component analysis (PCA) was employed to visualize the distribution of clinical characteristics in the two groups of JE patients (GP and PP) (11). The PCA analysis utilized packages such as “ggplot2”, “FactoMineR”, and “factoextra”. Correlation coefficient analysis between prognosis subtypes and clinical features was conducted using the R package “ggcorr”, and the results were visualized using “corrplot.mixed” (12).

## 3 Results

### 3.1 Machine learning results for Con vs. JE

Through the machine learning analysis using the R script, six major significantly risky factors were identified (*p* < 0.05) for distinguishing between the control group and patients with JE. These factors included WBC in CSF, total protein, N%, L%, RBC, and Cl^−^ concentration in blood. Changes in WBC in CSF and total protein in blood are commonly associated with viral infections. The significant changes in N% and L% are consistent with a previous proteomics study, which reported decreased levels of L%, M%, and increased levels of complement components as potential clinical markers for poor prognosis in JE (7). The statistical analysis based on mRS results further validated the significant changes in N% and L% in blood as indicators for the prognosis of JE.

### 3.2 Clinical characteristics of JE subgroup according to clinical outcome

A total of 38 severe adult patients with JE who were hospitalized were included in the analysis. The demographic and clinical characteristics of these patients are summarized in Table 1. The average age was 51.26 years (range: 35 to 65), and 24 patients (63.2%) were male. The most common signs and symptoms observed were fever (38/38, 100%), positive meningeal stimulation (31/38, 81.6%), disturbance of consciousness (30/38, 78.9%), headache (26/38, 68.4%), and mental symptoms (24/38, 63.2%). Among the patients, nine had a poor prognosis (mRs ≥ 3), including five deaths (mRs = 6), two with mRs = 3, and two with mRs = 4. All nine patients in the poor prognosis group exhibited disturbance of consciousness, and 7/9 (77.8%) required respiratory support and tracheal intubation. The Glasgow Coma Score (GCS) was significantly lower in the poor prognosis group compared to the good prognosis group (5.67 vs. 10.66, *p* = 0.001). A total of 25/38 cases (65.8%) were associated with pulmonary infection, and the ratio of pneumonia in the poor prognosis group was higher than that in the good prognosis group (88.9% vs. 58.6%, *p* = 0.126).

**Table 1:** Clinical characteristics of patients of JE grouped according to clinical outcome.

	Total (*N* = 38)	GP group (mRS ≤ 2) (*n* = 29)	PP group (mRS ≥ 3) (*n* = 9)	*p*-value
Sex, male, *n* (%)	24 (63.2)	21 (72.4)	3 (33.3)	0.052^c^
Age, median (IQR), years	51.26 [35–65]	49.03 [35–64]	58.44 [54–65]	0.294^b^
Close contact history with confirmed case, *n* (%)	31 (81.6)	25 (86.2)	6 (66.7)	0.322^c^
GCS score, median (IQR)	9.47 (5,14)	10.66 (8,14)	5.67 (5,8)	0.001^a^
Temperature, median (IQR), °C	39.12 (38.3,39.8)	39.07 (38.3,39.8)	39.31 (38.5,39.7)	0.321^a^
**Signs, *n* (%)**				
Fever	38 (100)	29 (100)	9 (100)	–
Headache	26 (68.4)	20 (69.0)	6 (66.7)	–
Weakness	20 (52.6)	14 (48.3)	6 (66.7)	0.462^c^
Nausea / Vomit	17 (44.7)	12 (42.9)	5 (55.6)	0.703^c^
Dizziness	15 (39.5)	12 (42.9)	3 (33.3)	0.711^c^
Respiratory failure	17 (44.7)	10 (34.5)	7 (77.8)	0.051^c^
Dyspnea	20 (52.6)	13 (44.8)	7 (77.8)	0.130^c^
Disturbance of consciousness	30 (78.9)	21 (72.4)	9 (100)	0.159^c^
Epilepsy	7 (18.4)	4 (13.8)	3 (33.3)	0.322^c^
Insanity	24 (63.2)	20 (69)	4 (44.4)	0.245^c^
**Symptoms, *n* (%)**				
Meningeal irritation sign	31 (81.6)	23 (82.1)	8 (88.9)	1
Hypermyotonia	9 (23.7)	7 (25)	2 (22.2)	1
Paralysis	19 (50)	12 (41.4)	7 (77.8)	0.124^c^
Pathological signs	18 (47.4)	12 (41.4)	6 (66.7)	0.260^c^
Image positive lesion	23 (60.5)	16 (55.2)	7 (77.8)	0.273^c^
**Coexisting disorder, *n* (%)**				
Hypertension	8 (21.1)	4 (14.3)	4 (44.4)	0.078^c^
Diabetes	3 (7.9)	1 (3.6)	2 (22.2)	0.141^c^
Coronary artery disease	3 (7.9)	1 (3.6)	2 (22.2)	0.141^c^
Pneumonia	25 (65.8)	17 (58.6)	8 (88.9)	0.126^c^

GCS, Glasgow Coma Scale; IQR, interquartile range.

a The data adheres to a normal distribution, *p*-values were calculated by the One-way ANOVA test.

b The data does not adhere to a normal distribution, *p*-values were calculated by the Wilcoxon rank-sum test.

c Enumeration data, *p*-values were calculated by the Chi-square test.

All the *p*-values indicate differences between patients with good prognosis and poor prognosis groups. *p* < 0.05 was considered statistically significant.

### 3.3 Laboratory results of JE subgroup

The laboratory results are presented in Table 2. The N% in the poor prognosis group (86.70% [IQR, 84.4–88.4%]) was significantly higher than that in the good prognosis group (81.50% [IQR, 77.5–84.3%]) (*p* = 0.01). The L% in the poor prognosis group (8.00% [IQR, 5.30–9.70%]) was significantly lower than that in the good prognosis group (11.10% [IQR, 9.50–14.90%]) (*p* = 0.032). The neutrophil-to-lymphocyte ratio (NLR) in the poor prognosis group (10.84 [8.06–17.58]) was significantly higher than that in the good prognosis group (7.11 [5.24–8.74]) (*p* = 0.01). CSF tests were performed on all 38 patients, and three cases had CSF WBC counts greater than 500 × 106/L, while one case had a CSF WBC count greater than 1,000 × 106/L. The latter patient was also diagnosed with suppurative meningitis and Kawasaki disease.

**Table 2:** Laboratory findings of patients with JE grouped according to the clinical outcome (median [IQR]).

	Total (*N* = 38)	GP group (mRS ≤ 2) (*n* = 29)	PP group (mRS ≥ 3) (*n* = 9)	*p*-value
**Peripheral blood test**				
White blood cell count, ×10^9^/L	11.94 [8.98–15.26]	10.55 [8.19–14.84]	15.15 [10.21–15.64]	0.171^a^
Red blood cell count, ×10^12^/L	4.43 [4.16–4.76]	4.50 [4.15–4.76]	4.34 [4.18–4.76]	0.723^a^
Neutrophil count, ×10^9^/L	9.15 [7.12–12.56]	8.27 [5.50–12.36]	12.03 [8.64–12.24]	0.061^a^
Neutrophil percentage, %	82.05 [78.08–86.28]	81.50 [77.50–84.30]	86.70 [84.40–88.40]	0.013^b^
Lymphocytic count, ×10^9^/L	1.18 [0.82–1.41]	1.22 [1.02–1.43]	0.83 [0.76–1.25]	0.083^b^
Lymphocytic percentage, %	10.75 [7.40–13.38]	11.10 [9.50–14.90]	8.00 [5.30–9.70]	0.032^a^
Neutrophil/Lymphocyte (NLR)	7.41 [6.04–11.49]	7.11 [5.24–8.74]	10.84 [8.06–17.58]	0.005^a^
Monocyte counts, ×10^9^/L	0.84 [0.52–1.17]	0.94 [0.53–1.20]	0.74 [0.24–1.07]	0.490^a^
Monocyte percentage, %	7.10 [5.33–8.40]	7.20 [6.40–8.80]	5.40 [2.50–7.20]	0.063^a^
Platelet count, ×10^9^/L	140 [107–164]	140 [114–160]	139 [96–170]	0.706^b^
Hemoglobin, g/L	135.00 [128–151]	135.14 [128–151]	131.78 [128–146]	0.663^a^
Aspartate aminotransferase, IU/L	22.5 [16.0–37.3]	24.0 [16.0–34.0]	75.0 [15.0–107.0]	0.757^b^
Alanine aminotransferase, IU/L	33.50 [18.3–53.0]	34.0 [15.0–53.0]	33.0 [20.0–51.0]	0.693^b^
Gamma-glutamyl transpeptidase, IU/L	26.0 [20.3–39.0]	26.0 [21.0–36.0]	20.0 [18.0–52.0]	0.536^b^
Serum potassium, mmol/L	3.68 [3.33–4.06]	3.75 [3.41–4.06]	3.39 [3.33–3.74]	0.157^a^
Serum sodium, mmol/L	135.5 [133.1–139.0]	136.3 [133.9–139.0]	133.1 [132.3–138.9]	0.439^a^
Serum chloride, mmol/L	101.65 [98.9–104.3]	101.7 [100.5–104.1]	98.9 [96.0–104.4]	0.374^a^
Blood glucose, mmol/L	7.05 [5.83–7.76]	6.50 [5.67–7.60]	7.74 [7.60–9.39]	0.004^a^
Urea nitrogen, mmol/L	5.9 [4.4–7.9]	5.9 [4.5–7.9]	5.4 [4.4–8.5]	0.420^a^
Creatinine, μmol/L	69.0 [56.8–76.8]	64.0 [55.0–76.0]	77.0 [68.0–83.0]	0.050^b^
Procalcitonin, ng/mL	0.20 [0.10–0.43]	0.11 [0.08–0.35]	0.27 [0.24–0.81]	0.487^a^
C-reactive protein, mg/L	34.01 [13.97–49.69]	33.38 [11.61–52.73]	36.14 [27.77–39.78]	0.779^a^
**Arterial blood gas test**				
pH	7.45 [7.43–7.47]	7.45 [7.42–7.47]	7.46 [7.44–7.47]	0.721^b^
Partial pressure of oxygen, mmHg	69.5 [60.8–79.0]	69.0 [60.5–79.0]	70.0 [66.6–76.0]	0.983^b^
Partial pressure of carbon dioxide, mmHg	32.0 [28.0–38.0]	32.0 [29.5–38.0]	29.0 [27.0–32.0]	0.200^b^
PaO_2_/FiO_2_, mmHg	246.0 [166.0–311.0]	246.0 [176.5–298.0]	246.0 [166.0–311.0]	0.787^a^
**CSF test**				
Open pressure, mmH_2_O	190.0 [130.0–220.0]	190.0 [120.0–202.5]	215.0 [150.0–250.0]	0.128^a^
CSF White cell count, ×10^6^/L	55.0 [24.0–107.5]	60.0 [20.0–100.0]	32.0 [30–120]	0.693^b^
CSF protein, mmol/L	680.5 [480.8–913.8]	679.0 [479.0–910.0]	830.0 [486.0–915.0]	0.188^a^
CSF glucose, mmol/L	3.51 [3.00–3.95]	3.30 [3.00–3.70]	4.00 [3.52–4.57]	0.046^a^
CSF chloride, mmol/L	122.2 [118.5–126.7]	121.5 [119.5–124.7]	124.9 [115.8–130.3]	0.358^a^

PaO_2_, partial pressure of oxygen; FiO_2_, fraction of inspired oxygen; IQR, interquartile range; CSF, cerebrospinal fluid. GP, good prognosis; PP, poor prognosis.

a The data adheres to a normal distribution, *p*-values were calculated by the One-way ANOVA test.

b The data does not adhere to a normal distribution, *p*-values were calculated by the Wilcoxon rank-sum test.

All the *p*-values indicate differences between patients with good prognosis and poor prognosis groups. *p* < 0.05 was considered statistically significant.

### 3.4 PCA result

**Figure 1:**
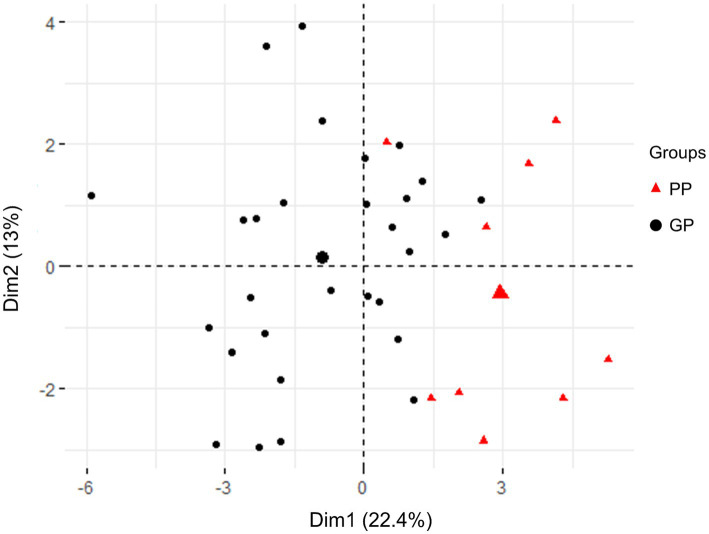
PCA score plot in all patients with JE based on clinical testing characteristics and clinical outcomes. The plots in GP (blank) and PP (red) group were apart from each other and could be easily differentiated. All plots within the group were clustered together, respectively.

To further validate the subgrouping results, PCA was employed to analyze the distribution of the two groups (GP and PP) based on their clinical characteristics. The results are presented in Figure 1. The PCA score plot demonstrated distinct clusters for patients with JE in the GP subgroup (blank) and the PP subgroup (red), indicating a differentiation based on clinical and laboratory features. Data from GP and PP was divided into two clusters by PCA (Figure 1). These results indicated that patients with poor prognosis could be distinguished from good prognosis through machine learning.

### 3.5 Correlation coefficient analysis for clinical JE subgroup

**Figure 2:**
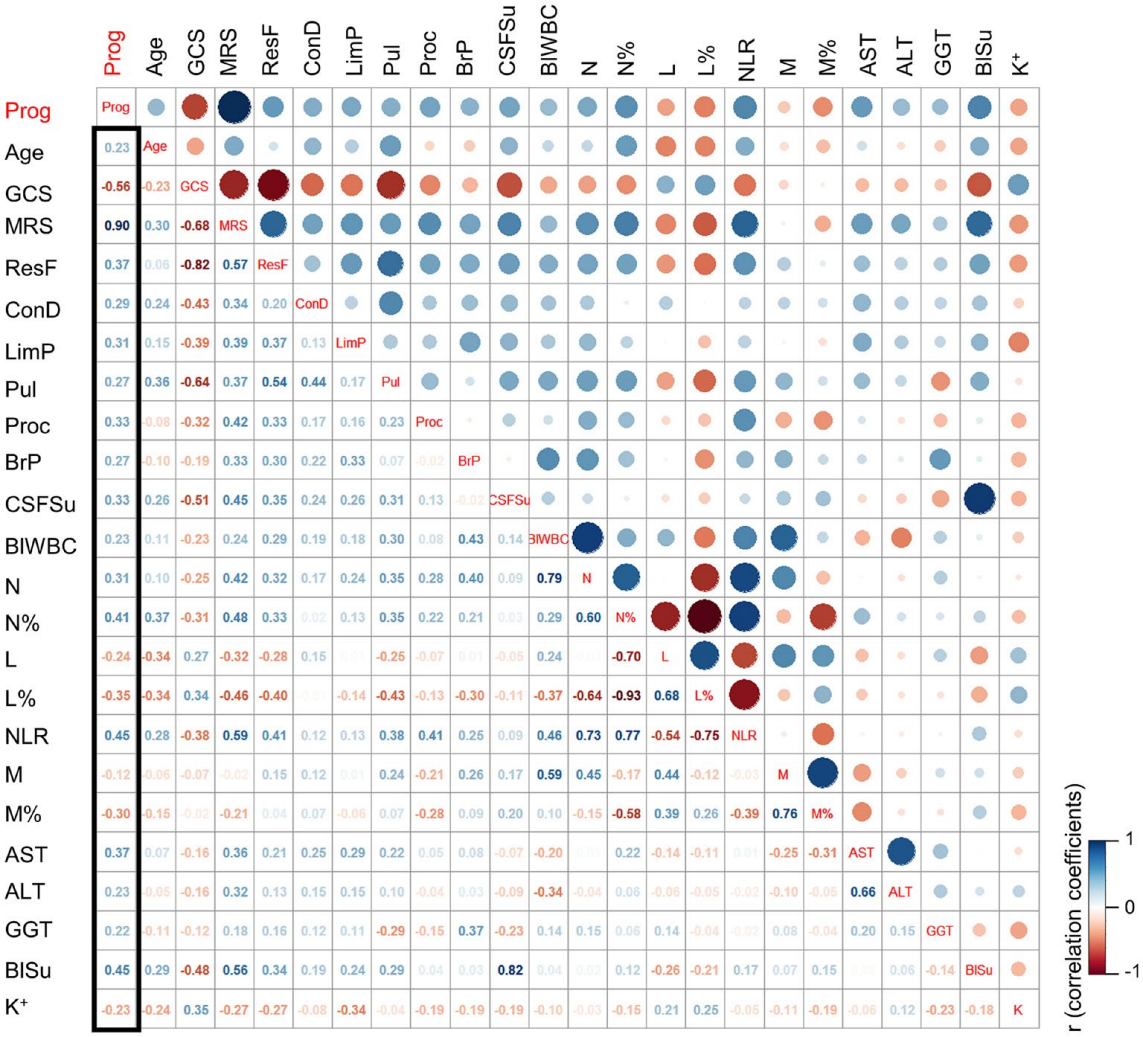
The correlation coefficient analysis for the selection of key clinical factors. The cut-off for clinical factors selection: *p* (PP vs. GP) ≤ 0.2. ResF, respiratory failure; ConD, consciousness disorder; LimP, limb paralysis; Pul, pulmonary infection; Proc, procalcitonin; BrP, open pressure for the brain; CSFSu, sugar in cerebrospinal fluid; BlWBC, blood WBC; N, neutrophils; N%, percentage of neutrophils; L, lymphocyte; L%, percentage of lymphocytes; M, monocyte; M%, percentage of monocyte; AST, the aspartate aminotransferase; ALT, the alanine aminotransferase; GGT, the gamma-glutamyl transferase; BlSu, blood sugar; K, potassium; Prog, prognosis; PP, poor prognosis; GP, good prognosis.

Correlation analysis was conducted to assess the relationship between clinical parameters and the prognosis of JE. The Pearson correlation coefficient (PCC) was calculated to determine the strength and direction of the correlations. The results revealed several clinical parameters that exhibited significant correlations with prognosis. The most closely related clinical parameters were GLU (PCC 0.45), NLR (PCC 0.45), N% (PCC 0.41), AST (PCC 0.37), respiratory failure (PCC 0.37), and L% (PCC −0.35), as depicted in Figure 2. These findings suggest that these parameters may serve as potential indicators for predicting the prognosis of JE.

Overall, the machine learning analysis identified significant risk factors for distinguishing between the control group and patients with JE. The clinical characteristics and laboratory results of the JE subgroup provided valuable insights into the prognosis of the disease. Furthermore, the PCA and correlation coefficient analysis further supported the differentiation of subgroups based on clinical and laboratory parameters and highlighted the potential predictive value of certain parameters for prognosis assessment in patient with JE.

## 4 Discussion

JE is a significant infectious disease that poses a serious threat to human health. Studies conducted in South Korea have reported a notable increase in the proportion of adults affected by JE in recent years (13). Additionally, a retrospective study spanning over 15 years in Gansu, China, found distinct clinical manifestations between children and adults (14). Children with JE commonly present with symptoms such as vomiting, irritability, hypersomnia, convulsions, and spasms. On the other hand, adults experience symptoms such as changes in blood pressure, pupil size, positive meningeal stimulation signs, and positive pathological reflexes (hypertonia and Babinski sign). These findings suggest that adults tend to exhibit more severe clinical manifestations and higher mortality rates. The differences in manifestations may be attributed to the absence of JE vaccination during childhood or the higher average age of onset (13). Therefore, it is crucial to identify risk factors for poor prognosis in adult patients with JE and develop personalized treatment plans that can provide more effective supportive care based on different subpopulations.

In our study, all patients presented with fever, and some also experienced symptoms such as headache, nausea, vomiting, and other manifestations of high cranial pressure. Consciousness and mental symptoms were prevalent among the patients. The machine learning analysis identified WBC in CSF, total protein in CSF, N%, L%, RBC in blood, and Cl^−^ as the key factors associated with JEV infection. Furthermore, the correlation coefficient analysis, using the clinical prognosis (mRS) as a grouping variable, revealed that increased NLR, N%, and decreased L% were risk factors associated with the clinical outcome of JE. Abnormal glucose metabolism and liver function were also closely associated with poor prognosis in JE. Additionally, the proportion of lung infections was higher in the poor prognosis group.

Our findings align with previous research, which demonstrated an increase in WBC, N%, and CSF WBC in severe JEV infection (15). JEV induces an inflammatory response characterized by the accumulation of various immune cells around the spleen, lymph nodes, and in the blood. This leads to increased WBC and neutrophil counts in the peripheral blood (16). Viral infections often result in increased lymphocyte counts, with or without elevated WBC levels. The potential pathogenesis underlying these observations involves nervous stress response, tissue damage response, immune response disorder, and secondary or mixed bacterial infections (2, 15, 16). In our study, 65.8% of the patients had lung infections, which may explain the increased percentage of WBC and neutrophils. Specifically, the poor prognosis group exhibited lower GCS scores, more severe disturbance of consciousness, and a higher likelihood of ventilator-associated pneumonia following tracheal intubation. Although WBC levels were outside the normal range, no statistically significant difference was observed between the poor prognosis and good prognosis groups. There were no significant differences in electrolyte, metabolite, and blood gas analysis between the two groups. Apart from bacterial infection, the increase in N% may be attributed to excessive cellular immune defense.

The correlation coefficient analysis demonstrated opposite trends in N% and L% with regard to prognosis. Increased NLR, N%, or decreased L% indicated a potential poor prognosis for patients with JE. These immunological findings highlight the important role of the innate immune system in the recovery from JEV infection. Previous research has reported elevated levels of certain components that are associated with the prognosis of JEV infection (7). Neutrophils express IgG Fc receptors, complement C3b, and C5a receptors on their surface. The upregulation of complement levels in the CSF of patients with JE may impact chemotaxis, thereby promoting and enhancing neutrophil phagocytosis. However, chemotaxis and excessive activation of neutrophils can be detrimental to the recovery from JEV infection. The overall condition of patients also plays a significant role in the prognosis of the disease, with liver function and glucose metabolism contributing to the impairment (17).

Once the virus enters the central nervous system, uncontrolled viral proliferation may occur (2). Proinflammatory cytokines can trigger immune cell infiltration and clearance of infectious viral factors. However, an excess of proinflammatory cytokines can lead to tissue damage and systemic inflammation (18). Previous studies have demonstrated that JEV infection, especially after the virus enters the CNS through the blood–brain barrier, triggers unrestricted viral proliferation, leading to a series of inflammatory reactions and activation of the systemic immune response (19). Overactivation of inflammatory cells can result in severe cytokine storms and tissue damage (20). This may explain why increased N% is a risk factor for poor prognosis in JE. Therefore, future treatments for severe JE may focus on regulating the innate immune response induced by the JE virus within a specific range, maintaining antiviral function while avoiding excessive inflammatory reactions.

Currently, two articles on the subject of JE and machine learning were reported, including one of our previous research. In a study performed by Tehmina et al., deep proteomic networks and machine learning techniques were employed to investigate the hypothesis regarding the presence of JE diagnostic protein signatures. However, it is worth noting that this study compared the CSF proteomics result from JE samples with other CNS infection patients, but without incorporating an appropriate control group. It is important to consider that patients with various infections, including those caused by other flaviviruses, may exhibit similar CSF protein expression patterns.

## 5 Limitations

### 5.1 The advantages and disadvantage of the method

Machine learning by stepwise algorithm is to automatically discover the major influential factors. Stepwise regression is useful when dealing with a large number of potential predictor variables. It automates the process of variable selection by sequentially adding or removing variables based on their statistical significance, which can be more efficient than manual selection (21). There are also some challenges existing in the stepwise method, one of the issues is that the remaining coefficients may be biased and need shrinkage. After each variable addition or deletion step, an evaluation metric is used to assess whether that step impacted fit. Giving primacy to individual fit, as is done with *p*-values and *R*^2^, when group fit may be the larger concern, can lead to misguided decision making (22). As the potential limitation of stepwise regression, we do statistical analysis first for the clinical results and input the clinical variables with significant differences, which helps to select the risky factor though the regression.

### 5.2 The limitations of the study

Despite the valuable findings of our study, several limitations should be acknowledged. Firstly, this was a single-center retrospective cohort study with a limited sample size. The inclusion of only severe cases and the absence of mild cases may introduce bias in the results. Secondly, the study lacked a comparison between severe and mild cases, which could have provided further insights. Lastly, there was a high proportion of patients lost to long-term follow-up, resulting in a lack of long-term prognostic outcomes.

## 6 Conclusion

The study identified significant risk factors associated with poor prognosis in adult patients with JE. These factors include WBC in CSF, total protein in CSF, N%, L%, RBC in blood, and Cl^−^. Correlation analysis further confirmed the association of increased NLR, N%, and decreased L% with adverse clinical outcomes. Additionally, abnormal glucose metabolism, liver function impairment, and a higher proportion of lung infections were observed in the poor prognosis group. The innate immune response appears to play a crucial role in the recovery from JEV infection, and maintaining a balanced immune response is crucial. Further research, including larger and prospective studies, is warranted to validate these findings and explore potential targeted therapies for severe JE.

In summary, our findings suggest that elevated serum NLR, N%, decreased L%, abnormal glucose metabolism, and liver function impairment may serve as potential prognostic indicators for severe adult patients with JE. However, further confirmation is required through prospective, multicenter randomized controlled trials with larger sample sizes. Additionally, conducting basic mechanisms research would provide deeper insights into these associations.

## Data availability statement

The original contributions presented in the study are included in the article/Supplementary material, further inquiries can be directed to the corresponding authors.

## Ethics statement

The studies involving humans were approved by the Ethics Committee of Lanzhou General Hospital. The studies were conducted in accordance with the local legislation and institutional requirements. The participants provided their written informed consent to participate in this study.

## Author contributions

PF, YH, YW, YH, and YL: conceptualization. YW, YL, MD, and HQ: data curation. YH, MD, and RL: formal analysis. YW, YH, YL, ZY, HQ, and RL: methodology. RY, PF, and YW: supervision. PF, YH, and MD: visualization. YW and MD: writing – original draft. RY and PF: writing – review and editing. All authors contributed to the article and approved the submitted version.
