# Supplementary material for: Machine learning reveals neutrophil-to-lymphocyte ratio as a crucial prognostic indicator in severe Japanese encephalitis patients
Source: Front Neurol. 2023 Dec 20;14:1242317. doi: 10.3389/fneur.2023.1242317 (PMC10765562; doi:10.3389/fneur.2023.1242317)
Supplement: Supplementary file 1 [file Image_1.pdf]

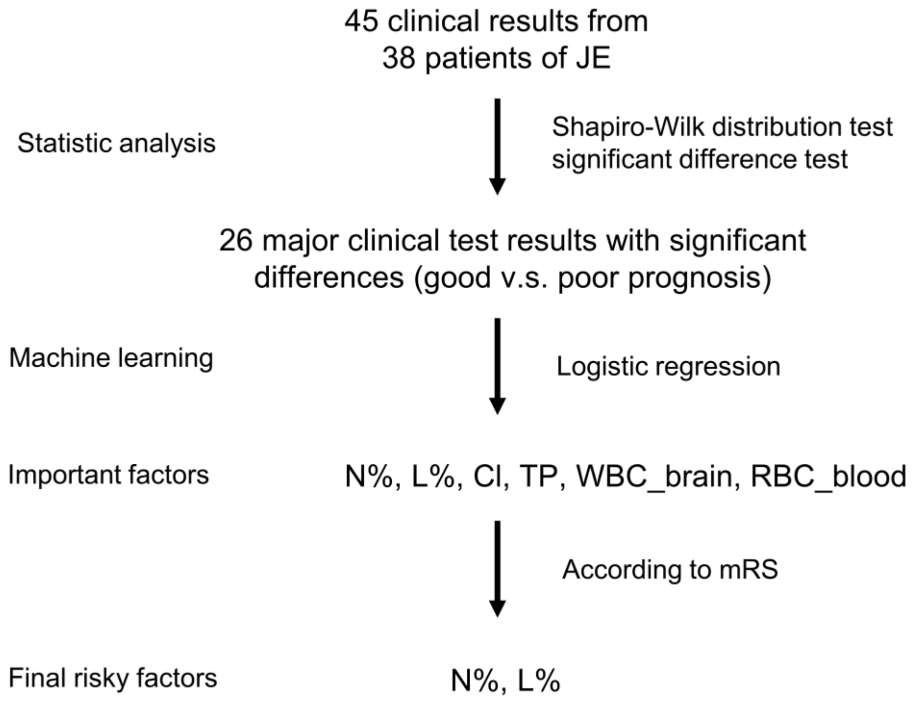

Supplementary figure1 The whole procedure of the machine learning analysis pipeline for JE Infection Risk Factors. The selection of risk factors was conducted through logistic regression, combining best subset selection and cross-validation approaches for model selection.
